# Poly(ADP-Ribose) Polymerase (PARP) Inhibitors for Cancer Therapy: Advances, Challenges, and Future Directions

**DOI:** 10.3390/biom14101269

**Published:** 2024-10-09

**Authors:** Denys Bondar, Yevgen Karpichev

**Affiliations:** Department of Chemistry and Biotechnology, Tallinn University of Technology (TalTech), Akadeemia tee 15, 12618 Tallinn, Estonia; debond@taltech.ee

**Keywords:** PARP inhibitors, poly(ADP-ribose) polymerase, anticancer agents

## Abstract

Poly(ADP-ribose) polymerases (PARPs) are crucial nuclear proteins that play important roles in various cellular processes, including DNA repair, gene transcription, and cell death. Among the 17 identified PARP family members, PARP1 is the most abundant enzyme, with approximately 1–2 million molecules per cell, acting primarily as a DNA damage sensor. It has become a promising biological target for anticancer drug studies. Enhanced PARP expression is present in several types of tumors, such as melanomas, lung cancers, and breast tumors, correlating with low survival outcomes and resistance to treatment. PARP inhibitors, especially newly developed third-generation inhibitors currently undergoing Phase II clinical trials, have shown efficacy as anticancer agents both as single drugs and as sensitizers for chemo- and radiotherapy. This review explores the properties, characteristics, and challenges of PARP inhibitors, discussing their development from first-generation to third-generation compounds, more sustainable synthesis methods for discovery of new anti-cancer agents, their mechanisms of therapeutic action, and their potential for targeting additional biological targets beyond the catalytic active site of PARP proteins. Perspectives on green chemistry methods in the synthesis of new anticancer agents are also discussed.

## 1. Introduction

Over the past several decades, the development of new anticancer drugs has increasingly focused on targeting specific molecular pathways to exploit the unique vulnerabilities of cancer cells. This strategy has led to a significant rise in the identification of tumor-specific molecular aberrations for drug targeting, improving survival rates with biomarker-matched therapies across various cancer types. Consequently, molecular pathology now plays a crucial role in tumor diagnosis, prognosis, and guiding therapeutic decisions [1]. One such target is the DNA damage response (DDR) mechanism that is critical for maintaining genomic stability and preventing cancer. The development of DDR inhibitors, particularly targeting poly(ADP-ribose) polymerase 1 (PARP1), offers new strategies for enhancing the effectiveness of traditional cancer treatments and improving patient outcomes. PARP1, an enzyme involved in various cellular processes from DNA repair to cell death, is crucial for recognizing and responding to DNA breaks, positioning it as an essential first responder to DNA damage [2,3].

**Figure 1:**
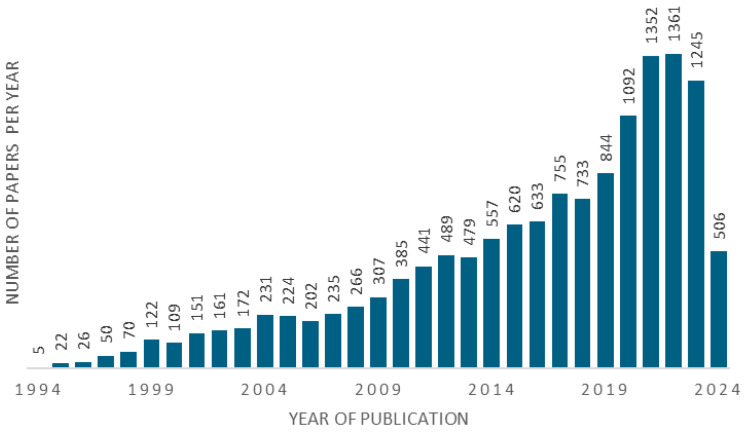
Bibliometric data on peer-reviewed documents published on ‘PARP inhibitors’ according to the Clarivate Analytics Web of Science (WoS, all databases) for the years 1994–2024; the inquiry was made on 16 July 2024.

PARP1 inhibitors are a promising class of drug candidates (see Figure 1) that target DNA repair deficiencies in cancer cells, especially those with BRCA1/2 mutations [4,5,6]. Clinical trials have shown the effectiveness of PARP1 inhibitors such as olaparib **1**, rucaparib **2**, and niraparib **3** in improving patient survival in BRCA-mutant breast and ovarian cancers. The various compounds analyzed in this study, along with their codes, are detailed in Figure S1. These inhibitors block the activity of PARP1, leading to the accumulation of DNA damage and ultimately cell death, especially in tumors deficient in homologous recombination (HR) repair—a mechanism known as synthetic lethality [7,8]. This approach illustrates how knowledge of the cell pathologies of tumors can lead to more effective and targeted treatments. However, drug resistance often develops, requiring combination with chemotherapy, immune checkpoint inhibitors, or anti-angiogenesis drugs to enhance treatment efficacy and overcome resistance.

Several studies emphasize the importance of targeting specific molecular pathways in cancer therapy. Advances in CRISPR-Cas screening technology have greatly enhanced our understanding of drug resistance mechanisms by identifying genetic alterations and pathways that contribute to resistance [9]. Technology allows for systematic gene editing, revealing mutations, alternative signaling pathways, and regulation of drug efflux. These discoveries facilitate the development of novel, targeted therapies and enhance personalized medicine by integrating findings with patient-focused models for more effective treatments [2,9,10,11,12]. Additionally, advances in gene profiling have facilitated the development of precision medicine, allowing for the identification of actionable genetic mutations in patients’ tumor samples, thereby improving prescribed therapeutic strategies [1,13].

When DNA breaks occur, especially those caused by alkylating agents and radiation, PARP1 binds to the damaged sites via its DNA-binding zinc finger domains [14]. Once bound, PARP1 produces oligo- or poly(ADP)-ribose chains, which facilitate chromatin decondensation at the damage site. This step is crucial, as it lets repair enzymes access the dysfunctional DNA and fix it [15]. The PARP inhibitors are critically important in rapidly proliferating tumor cells with defective DNA, preserving gene stability [16]. While PARP1 helps overcome minimal genotoxic damage, severe damage can trigger apoptosis or necrosis due to PARP overactivation, leading to cell death if caspase activation is inadequate [17].

The involvement of PARP1 in cancer development is well studied [18]. Loss of PARP1 interrupts DNA repair processes, inhibits the transcription of genes involved in DNA replication and cell cycle regulation, and can contribute to genomic instability [19]. Consequently, elevated PARP1 expression is observed in various tumors, including melanomas [20], lung, and breast tumors [21]. In these types of cancer, high levels of PARP1 are often associated with low survival rates and more aggressive tumor phenotypes. This increased lethality likely occurs since increased PARP1 levels facilitate DNA damage repair, allowing oncogenic cells to survive and proliferate despite inherited instability in their genome.

The role of PARP1 in cancer has made it a significant target for therapeutic action. Targeting PARP1 with inhibitors can expose tumor cells to DNA-damaging agents, making them more susceptible to chemotherapy and radiation. This approach relies on the idea of synthetic lethality, where the inhibition of PARP1 in cancer cells with defective DNA repair mechanisms (such as BRCA1/2 mutations) leads to cell death, while normal ones with intact DNA repair pathways are less affected [9,22].

## 2. History of Development of PARP Inhibitors

**Figure 2:**
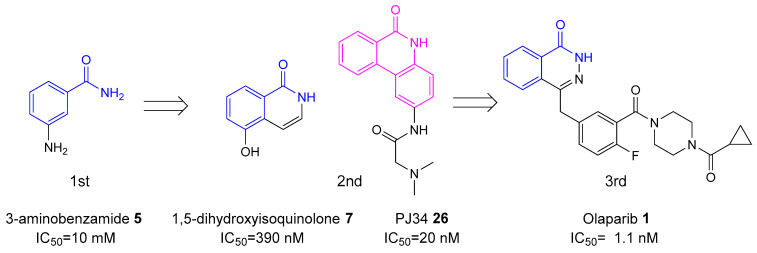
Sequential development of first-, second-, and third-generation PARP inhibitors; the nicotinamide moiety is highlighted in blue; the phenanthridinone core in PJ34 **26** structure is highlighted in pink.

The history of PARP inhibitors’ development can be divided into three sequential generations of agents (Figure 2), each marked by improvements in drug efficacy, specificity, and clinical viability [23].

**Figure 3:**
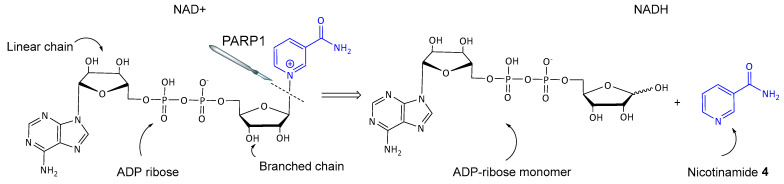
Mechanism of PARP1-mediated ADP-ribose polymerization from NAD+ to NADH. The cleavage of the bond indicated in the picture liberates the nicotinamide **4** moiety, which is highlighted in blue, and the ADP-ribose monomer is incorporated into linear or branched polymers.

The first generation of PARP inhibitors, developed during the late 1970s and early 1980s, included nicotinamide analogs since nicotinamide, the second product of the PARP1-catalyzed reaction (see Figure 3), causes moderate inhibition of PARP1 [24,25]. These early discovered inhibitors, such as 3-aminobenzamide (3-AB) **5**, demonstrated the potential to enhance the cytotoxic effects of DNA-damaging agents, but were not as selective and 1000 times less potent than newer inhibitors [26]. Nevertheless, they provided important insights into the role of PARP1 in DNA repair and tumor survival, giving incentive to the development of more potent and selective PARP inhibitors in the following decades [27]. Moreover, their practical use was restrained due to the high concentrations required to achieve therapeutic effects and their nonspecific inhibition of other cellular pathways [28].

**Figure 4:**
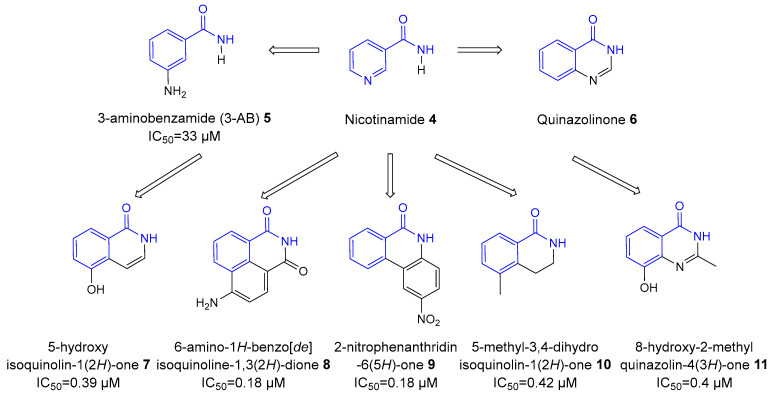
Structures and inhibitory potency towards PARP1 enzyme of various second-generation PARP inhibitors derived from 3-aminobenzamide **5**; the nicotinamide moiety is highlighted in blue.

In the 1990s, the development of more effective second-generation PARP inhibitors (Figure 4), based on quinazoline analogs, marked significant progress [29]. These included isoquinolines [30], quinazolinones, and phthalazinones [31]. Drug candidates such as 5-methyl-3,4-dihydroisoquinolin-1(2*H*)-one (PD128763) **10** and 8-hydroxy-2-methylquinazoline-4(3*H*)-one (NU1025) **11** were approximately 50 times more effective than 3-AB **5** in inhibiting the PARP1 enzyme [32]. These inhibitors contained a carboxamide group in their scaffolds, which formed strong and stable hydrogen bonds within the catalytic active site (CAS) of PARP1, greatly enhancing their affinity and specificity. The addition of this carboxamide group allowed for precise interactions with key proteins in the active site, such as Glu988, which plays a crucial role in the function of PARP1. This strategic modification not only improved the inhibitors’ potency by stabilizing their binding but also increased their selectivity by effectively targeting the PARP1 enzyme, thus minimizing off-target effects and enhancing drug efficacy [33].

Further research led to the creation of third-generation PARP inhibitors, with rucaparib **2** being the first characterized representative [34]. Rucaparib **2**, a potent PARP1 inhibitor, not only blocks PARP1 catalytic activity but also traps PARP1 on damaged DNA, significantly enhancing cytotoxic effects in HR-deficient cells. Clinical development of rucaparib included extensive trials demonstrating its efficacy as a single-agent therapy and as an adjunct to post-platinum chemotherapy in ovarian cancer. Its approval by regulatory agencies has paved the way for its use in other cancer types, including those with different HR gene defects, establishing rucaparib **2** as a pillar in the advancement of PARP inhibitor therapies [35,36].

**Figure 5:**
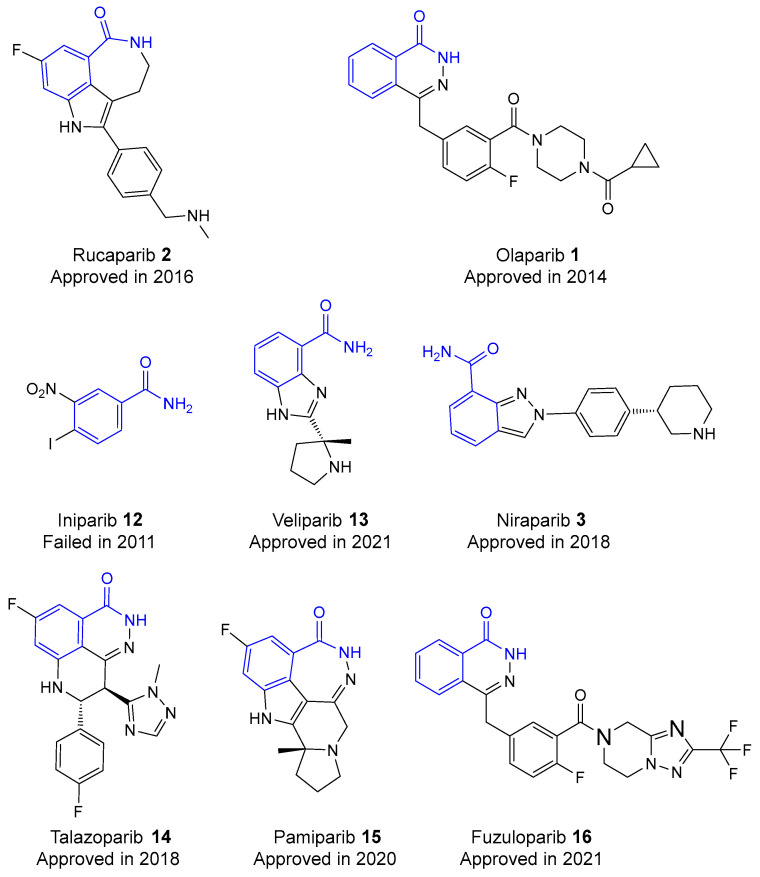
Structures of various third-generation PARP inhibitors and their approval rates; the nicotinamide moiety is highlighted in blue.

Other hit compounds (see Figure 5), including olaparib **1**, iniparib **12**, veliparib **13**, niraparib **3**, talazoparib **14**, pamiparib **15**, and fuzuloparib **16**, are currently undergoing clinical trials. Olaparib (Lynparza) **1** was used in the US and Europe for ovarian cancer treatment [37]. It also shows potential for use in acute ischemic stroke, TBI, septic shock, and severe asthma due to its protective effects in preclinical trials [38]. Veliparib has been given FDA Fast Track approval for developed lung cancer when combined with chemotherapies [39]. Niraparib **3** is under FDA Fast Track review for recurrent platinum-sensitive ovarian, fallopian tube, or primary peritoneal cancer [40]. Talazoparib **14** is in Phase III trials for breast cancer [41]. These facts imply the broad research to evaluate the efficacy of these PARP inhibitors in both oncological and non-oncological diseases [38]. Third-generation inhibitors are designed to be more potent and selective, with improved pharmacokinetic properties. Crystallographic studies and docking simulations showed that this generation forms multiple critical hydrogen bonds with the active sites of PARP1, notably involving residues such as Ser904, Gly863, Tyr896, and Trp861 in PARP1. Moreover, these compounds exhibited superior antiproliferative activities against various cancer cell lines, strong anti-angiogenesis activity, and the ability to increase intracellular ROS levels, further implying their therapeutic potential and improved pharmacokinetic profiles [42].

Over the past 10 years, clinical trials have resulted in the regulatory approval of several PARP inhibitors for treating breast and ovarian cancer [35]. The US Food and Drug Administration (FDA) has approved four third-generation PARP inhibitors—olaparib **1** (Lynparza; AstraZeneca, Cambridge, United Kingdom), rucaparib **2** (Rubraca; Clovis Oncology, Boulder, CO, USA), niraparib **3** (Zejula; GSK, London, United Kingdom), and talazoparib **14** (Talzenna; Pfizer, New York, NY, USA)—for clinical use as single-drug agents [43]. These medications share a similar chemical structure that allows them to compete with NAD for PARP binding [44]. Veliparib **13** is also expected to receive approval soon, based on positive outcomes from Phase III trials [45]. Meanwhile, some PARP1 inhibitors have been withdrawn from trials due to significant toxicity and adverse side effects [46]. A notable example is iniparib **12** (BSI-201), which failed in a Phase III trial for non-small-cell lung cancer and a Phase II trial for platinum-resistant ovarian cancer [47].

## 3. Base Excision Repair (BER) Mechanism

**Figure 6:**
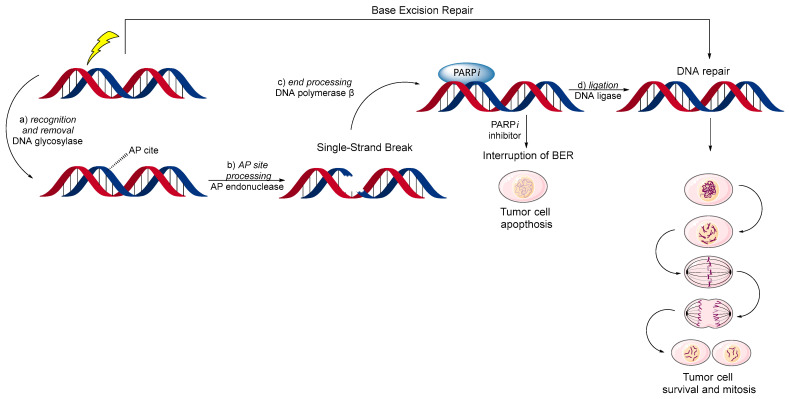
Interruption of normal BER mechanism of DNA repair by PARP inhibitors. (**a**) recognition and removal, when a DNA glycosylase enzyme recognizes and removes the damaged base, creating an abasic site (AP site); (**b**) AP site processing, where an AP endonuclease cleaves the DNA backbone at the abasic site, producing a single-strand break (SSB) with 3′-hydroxyl and 5′-deoxyribose phosphate termini; (**c**) end processing, which involves addition by DNA polymerase β of a new nucleotide to the 3′ end and removal of the 5′-deoxyribose phosphate group; (**d**) ligation, where DNA ligase seals the nick, completing the repair process.

Base excision repair (BER) is a cellular mechanism that repairs damaged DNA throughout the cell life cycle (see Figure 6). It mostly corrects small base lesions that usually do not significantly distort the DNA helix structure. The BER process involves several key steps [48].

PARP1 and 2 subtypes play a critical role in BER by detecting SSBs and delivering the necessary repair proteins to the site of damage [49]. Inhibition of PARP1/2 interrupts the BER process, leading to the persistence of SSBs. These SSBs can escalate into more lethal double-strand breaks (DSBs), particularly during DNA replication [50].

## 4. Two Models of Action of PARP Inhibitors

PARP inhibitors exhibit their anticancer effects by interrupting DNA repair pathways and enhancing the cytotoxic effects of DNA-damaging agents.

Interruption of DNA repair by the inhibitors occurs through direct inactivation of the repair system and preservation of spontaneous single-strand breaks (SSBs), which can lead to DSBs. Specifically, PARP inhibitors prevent PARP1 from binding to DNA breaks and initiating repair, causing SSBs to persist and eventually transform into DSBs during DNA replication [51]. DSBs in DNA can be repaired through error-free homologous recombination (HR) or error-prone non-homologous end joining (NHEJ) [52]. Tumor cells with HR deficiencies, such as those with BRCA1 mutations, are particularly sensitive to inhibition of poly(ADP-ribose) polymerase. These cells struggle to repair DSBs effectively, leading to genomic instability and cell death. These BRCA1 mutations impair HR, making these cancers initially sensitive to PARP inhibitors but also capable of developing resistance through mechanisms that restore HR functionality [53].

**Figure 7:**
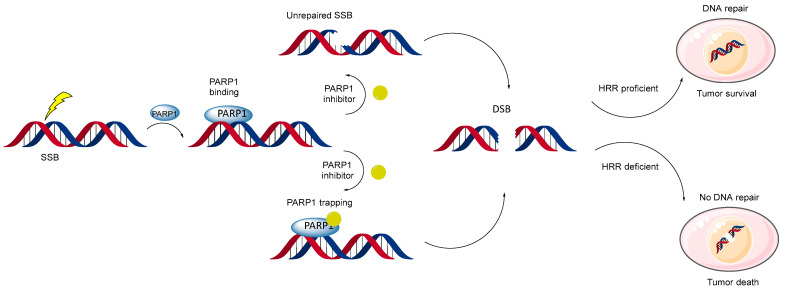
Two models of PARP1 action: (top) PARP inhibitors prevent SSB repair, leading to DSBs; (bottom) PARP inhibitors trap PARP1 on DNA, creating cytotoxic PARP1-DNA complexes in cells unable to repair DSBs effectively. Both models result in cell death in HR-deficient tumors (e.g., BRCA mutations), while HR-proficient cells using BRCA1/2 survive.

Several models explain the direct action of PARP inhibitors on DNA repair mechanisms (Figure 7) [54,55]. The most popular one suggests that PARP inhibitors trap PARP1 at damaged DNA sites, preventing it from detaching and blocking the start of repair. Studies support this, showing that physical trapping of PARP1 is essential for the inhibitors’ action [56]. Recent findings reveal that PARP inhibitors also trap PARP2, extending the lifetime of DNA damage-induced foci [57]. This trapping is independent of PARylation activity and requires specific mutations in the WGR and catalytic domains. Enhanced trapping of PARP2, especially in PARP1-deficient cells, underscores the distinct roles of PARP1 and PARP2 in DNA repair. These findings highlight how PARP inhibitors effectively target HR-deficient cancer cells, not only by inhibiting enzymatic activity but also by physically trapping these proteins at DNA damage sites, hampering repair, and leading to increased cell death in cancers with homologous recombination deficiencies [58]. Another model highlights that in the presence of PARP inhibitors, mutant BRCA1 tends to accumulate less of the enzyme at DNA damage sites, further inactivating the repair process. Additionally, PARP inhibitors may activate an alternative NHEJ repair system in HR-deficient cells [4]. This activation leads to increased genomic instability and cell apoptosis, particularly in cells with BRCA mutations that already have decreased HR repair capabilities [51]. This alternative pathway’s error-prone nature contributes to the lethal accumulation of DNA damage. These models collectively explain the efficacy of PARP inhibitors in targeting cancer cells with specific genetic deficiencies, particularly those involving BRCA1/2 mutations, thereby providing a rationale for their use in targeted cancer therapies.

Radiation therapy and many chemotherapeutic approaches rely on inducing DNA damage [45,59]. PARP inhibitors enhance these treatments by acting as chemo- and radiosensitizers [60,61]. By inhibiting PARP enzymes, these inhibitors interrupt the repair of SSBs in DNA, leading to the formation of DSBs during DNA replication. This mechanism is particularly effective in tumor cells with homologous recombination (HR) deficiencies, such as those with BRCA mutations, as these cells are unable to efficiently repair DSBs, leading to increased tumor death. Several studies have shown that PARP inhibitors increase the cytotoxicity of DNA-methylating agents such as temozolomide **17** and topoisomerase I inhibitors such as topotecan **18** [60,62,63].

For instance, the combination of temozolomide **17** with PARP inhibitors has been able to enhance the drug’s cytotoxic effects in cancer cells, making it a more potent therapeutic solution [64,65]. Additionally, PARP inhibitors act as radiosensitizers [66]. They enhance the effectiveness of ionizing radiation by preventing the repair of SSBs, which are converted into DSBs during replication, thus amplifying the DNA damage induced by radiation therapy. This combination has been studied in various clinical trials, indicating better outcomes in patients receiving both treatments consequently [67,68,69]. For example, clinical trials that combined veliparib **13** with brain radiation therapy for brain metastases and olaparib **1** with radiotherapy in prostate cancer patients have shown promising results [70].

## 5. Synergy of PARP1 Inhibitors and DNA Methylating Agents

**Figure 8:**
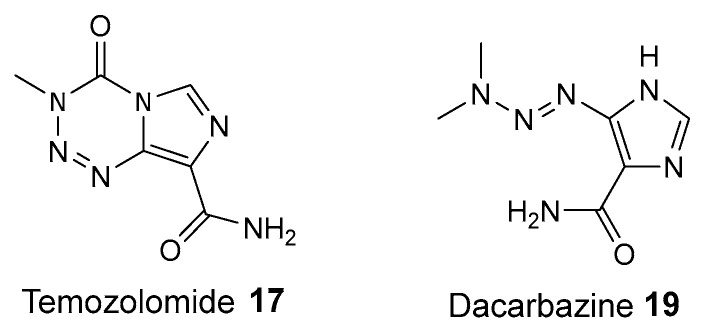
Structures of DNA-methylating agent temozolomide **17** and dacarbazine **19**.

Studies using first-generation PARP inhibitors such as 3-AB **5** have shown that these inhibitors enhance the effects of DNA-methylating agents such as temozolomide (TMZ) **17** and dacarbazine (DTIC) **19** (see Figure 8), used in treating brain tumors and melanomas [71,72]. 3-AB **5** inhibits PARP1, thus the PARP inhibitor disrupts the BER pathway, preventing SSB repair. This leads to the accumulation of DSBs during replication, increasing genomic instability and cell death.

Combining 3-AB **5** with DNA-methylating agents has been shown to potentiate their anti-tumor effects, making cancer cells more susceptible to DNA damage [73]. This synergy extends to newer, more potent PARP inhibitors such as olaparib **1**, which have been tested in clinical trials with TMZ **17** for various cancers, including glioblastoma and small cell lung cancer, with promising results [74,75]. However, the development of resistance to PARP inhibitors through mechanisms such as secondary BRCA mutations or upregulation of drug efflux transporters remains a challenge [76]. Addressing these resistance mechanisms is crucial for maximizing the therapeutic potential of PARP inhibitors in combination therapies [77]. Early research showed that combining PARP inhibitors with temozolomide **17** increased TMZ’s cytotoxicity by four to seven times at lower concentrations. In the Phase II trial, the PARP inhibitor veliparib **13** was used with TMZ **17** to test this effect in metastatic breast cancer patients. Patients received veliparib **13** (30–40 mg twice daily, days 1–7) and TMZ **17** (150 mg/m^2^, days 1–5) in a 28-day cycle. The study included 41 patients without BRCA deficiencies and 21 volunteers having BRCA1/2 mutations. Results showed a higher objective response rate (ORR) among BRCA1/2 mutation patients (23%) compared to non-affected ones (0%). Notably, patients with BRCA1/2 mutations without prior platinum therapy had an ORR of 32% and a median progression-free survival (PFS) of 6.2 months. This confirms the enhanced cytotoxicity of the veliparib **13** and TMZ **17** combination, particularly in BRCA-associated metastatic breast cancer [78]. This synergistic effect is likely due to the accumulation of DSBs during replication. In vivo studies have further supported the efficacy of joint administration of PARP inhibitors with DNA-methylating agents, leading to the start of clinical trials exploring these effects [79].

## 6. Synergy of PARP1 Inhibitors and Topoisomerase I (TOP1) Inhibitors

**Figure 9:**
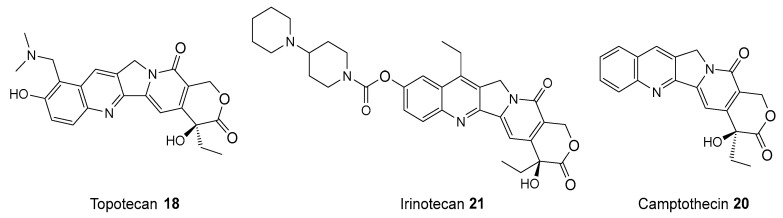
Structures of TOP1 inhibitors used.

Topoisomerase I inhibitors, such as camptothecin **20** (see Figure 9), are used to treat various tumors by stabilizing the cleavable TOP1-DNA complex, which leads to the accumulation of DNA breaks and ultimately induces cell death [80]. In the study, several PARP inhibitors, including veliparib **13** and olaparib **1**, were examined for their potential to enhance the efficacy of TOP1 inhibitors [81]. For instance, in one of the experiments, the combination of irinotecan **20** with olaparib **1** was tested. Irinotecan **21**, a prodrug that is converted into the active metabolite SN-38, was administered at 200 mg/m^2^ every three weeks, alongside olaparib **1** at a dose of 50 mg every day for 21 days. This combination therapy aimed to exploit a specific weakness in cancer cells. It targeted DNA damage from TOP1 inhibitors and blocked PARP. The idea was simple: stopping PARP would prevent repair of DNA breaks caused by TOP1 inhibitors, leading to more cancer cell deaths. However, the study faced serious challenges by experiencing major side effects, such as low blood cell counts and diarrhea. These made it crucial to manage doses carefully, but the findings also showed the need to improve treatments to ensure they are both effective and tolerable [13].

PARP1 inhibitors enhance the cytotoxic effects of TOP1 inhibitors by preventing the repair of TOP1-induced damage [82]. For example, the PARP inhibitor rucaparib **2** was used in combination with the DNA-methylating agent temozolomide **17**. Pre-clinical data showed that rucaparib **2** significantly increased the cytotoxic activity of temozolomide **16**. Rucaparib **2** inhibits PARP1, a crucial enzyme in repairing DNA single-strand breaks. By blocking this repair, rucaparib **2** leads to an accumulation of DNA damage in cancer cells, resulting in enhanced cell death. This combination was tested in a phase I clinical study, where rucaparib **2** and temozolomide **17** were administered to patients with advanced solid tumors, demonstrating significant tumor PARP inhibition and notable clinical results [83].

**Figure 10:**
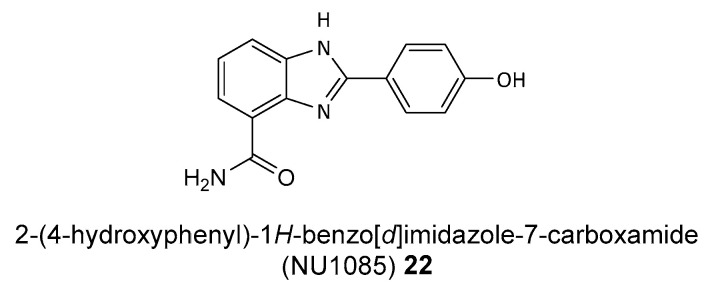
Structures of PARP inhibitor NU1085 **22**.

Studies have shown that PARP inhibitors such as NU1025 **11** and 2-(4-hydroxyphenyl)-1*H*-benzo[*d*]imidazole-7-carboxamide (NU1085) **22** (see Figure 10) significantly enhance the exposure of tumor cells to topoisomerase I inhibitors, such as topotecan **18** (see Figure 9). These findings highlight the potential for using a combination of PARP and TOP1 inhibitors to improve therapeutic outcomes in cancer treatment. By inhibiting PARP1, these inhibitors prevent the repair of DNA damage induced by topotecan **18**, leading to increased cytotoxicity in tumor cells. In vivo experiments have demonstrated that combination of PARP with TOP1 inhibitors results in improved therapeutic efficacy [13].

## 7. Synergy of PARP1 Inhibitors and Radiotherapy

Ionizing radiation, encompassing X-rays, gamma rays, and particles such as alpha and beta particles, inflicts damage to cellular DNA, potentially inducing cell death through SSBs or more severe DSBs. Initially, ionizing radiation induces SSBs, which cells typically repair, turning them into DSBs. The accumulation of unrepaired DSBs overwhelms cancer cells’ repair mechanisms, ultimately inducing cell death. The pre-treatment with olaparib **1** enhanced the sensitivity of cancer cells to ionizing radiation-induced DNA damage, leading to increased cell death compared to cells treated with radiation alone or olaparib **1** alone [64]. Early studies have demonstrated that PARP inhibition can lead to the radiosensitization of mammalian cells, thereby enhancing the effectiveness of radiation therapy [68]. For instance, veliparib **13** has been extensively studied for its radiosensitizing properties. One notable experiment involved treating non-small cell lung cancer (NSCLC) cell lines, A549 and Calu-6, with veliparib **13**. The study revealed that pretreatment with veliparib **13** at concentrations of 1 μM and 5 μM increased the radiosensitivity of both cell lines in a dose-dependent manner when exposed to varying doses of ionizing radiation (0, 2, 4, and 6 Gy). Specifically, the survival enhancement ratios (SER) at 1 μM were found to be 1.3 for A549 and 1.5 for Calu-6, which further increased to 1.6 and 1.8, respectively, at the 5 μM concentration [84]. Preclinical studies have shown that combining PARP inhibitors with ionizing radiation significantly enhances anti-tumor activity. Again, veliparib **13** was used to increase the radiosensitivity of human medulloblastoma cells. In experiments with D425 and D283 cell lines, veliparib **13** delayed DNA damage repair, increased DNA damage markers, and reduced colony formation. In an orthotopic xenograft model, mice with intracranial D425 medulloblastoma xenografts treated with veliparib and TMZ **16** multifractionated craniospinal irradiation (CSI) exhibited reduced tumor growth, increased apoptosis, and significantly improved survival compared to CSI alone [85]. These findings indicate the potential for combining PARP inhibitors with radiotherapy to enhance cancer treatment efficacy by leveraging their ability to intensify DNA damage and trigger cell death in tumor cells.

## 8. PARP1 Inhibitors with Cytostatic Drugs

**Figure 11:**
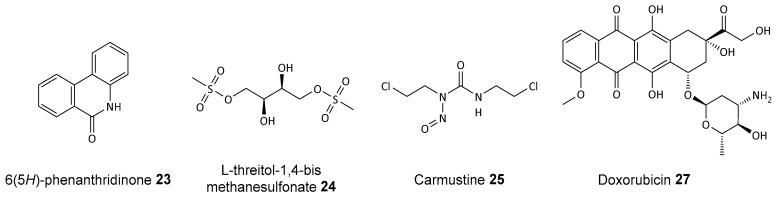
Structures of PARP inhibitor PHD **23** and DHB **24**, an alkylating agent, used jointly. Carmustine **25** and doxorubicin **27** are non-selective anticancer drugs.

PARP inhibitors have also been shown to enhance the effects of other cytostatic drugs. For example, in a study involving human and rat ovarian tumor cell lines, the PARP inhibitor 6(5*H*)-phenanthridinone (PHD) **23** (see Figure 11) was tested for its ability to enhance the cytotoxicity of various chemotherapeutic agents. Specifically, 6(5*H*)-phenanthridinone **23** was found to enhance the cytotoxicity of the alkylating agent L-threitol-1,4-bismethanesulfonate (DHB) **24** in the cisplatin-resistant rat ovarian tumor cell line O-342/DDP. However, it did not increase the cytotoxicity of carmustine **25** in the tested cell lines [86], notably PJ34 **26**, at concentrations higher than those required for PARP inhibition, significantly increasing the cytotoxicity of doxorubicin **27**. The experiments demonstrated that treatment with PJ34 **26** at concentrations of 20–30 μM eradicates HeLa cells, which are resistant to other therapies. This is achieved by causing irreversible anomalies in their mitotic spindle structure. This leads to mitotic catastrophe and cell death, thereby enhancing the efficacy of doxorubicin **27**.

**Figure 12:**
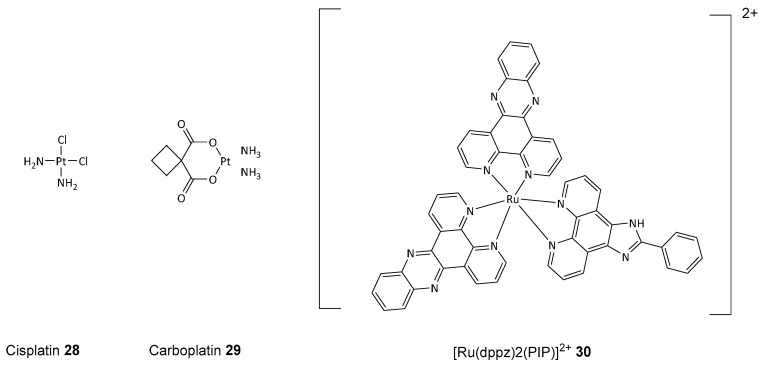
Pt- and Ru-based anticancer drugs, cisplatin **28** and carboplatin **29**, and [Ru(dppz)_2_(PIP)]^2+^ **30**.

This selective cytotoxicity was exclusive to cancer cells, as PJ34 **26** did not affect the viability of healthy proliferating cells in similar conditions [87]. Additionally, the synergistic action of PARP inhibitors with platinum compounds, such as cisplatin **28** and carboplatin **29** (see Figure 12), has led to clinical trials exploring these combinations. For example, PJ34 **26** enhances cisplatin’s effects in triple-negative breast and liver cancers. This causes permanent DNA damage and increased cell mortality, restricting tumor expansion.

Moreover, ruthenium complexes such as [Ru(dppz)2(PIP)]^2+^ **30** demonstrated synergy with olaparib **1**, increasing DNA damage and cell death in TNBC cells while maintaining high tumor selectivity. Similarly, combining carboplatin **29** with olaparib **1** in high-grade serous ovarian cancer cells showed increased DNA double-strand breaks and tumor growth inhibition, effective even in BRCA2-mutated cancers. Clinical trials have confirmed the efficacy of olaparib **1** with carboplatin-paclitaxel in high-grade and recurrent ovarian cancer, improving progression-free survival with manageable toxicity [88].

## 9. Dual PARP and HDAC (Histone Deacetylases) Inhibitors

**Figure 13:**
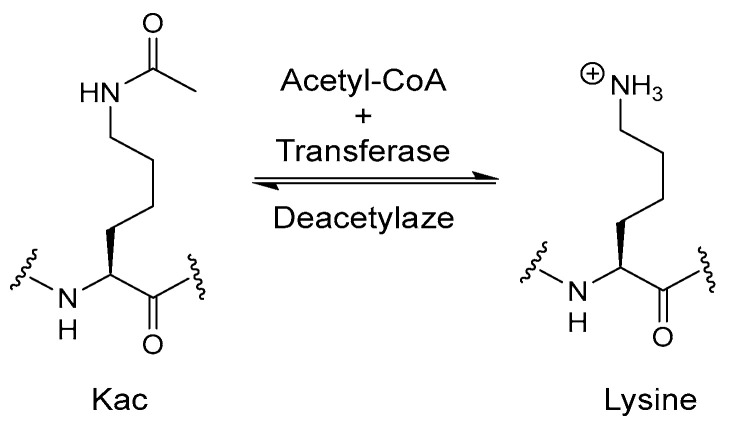
Normal function of HDAC in cell.

HDACs are enzymes that remove acetyl groups from histone proteins (see Figure 13), leading to chromatin condensation and transcriptional repression [89]. Inhibiting HDACs can reactivate silenced genes involved in cell cycle regulation and apoptosis, thus exhibiting antitumor effects. The rationale for dual inhibition lies in the synergistic effects of targeting both DNA repair and epigenetic regulation [90]. Compounds that inhibit both PARP and HDAC can potentiate DNA damage while simultaneously reactivating apoptotic pathways, leading to enhanced cancer cell killing [91].

**Figure 14:**
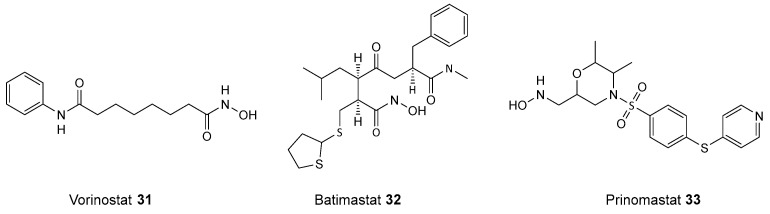
Structures of HDAC inhibitors based on hydroxamic moiety.

Hydroxamic acids, known for their α-nucleophilic properties, hold significant pharmaceutical potential, particularly as inhibitors of metal-dependent enzymes such as HDAC. They became a prominent class of HDAC inhibitors due to their superior ability to chelate zinc ions in the active site of HDACs, clearly demonstrated by the wide adoption of vorinostat **31**, batimastat **32**, and prinomastat **33** (see Figure 14) [92].

However, the stability of hydroxamic acid-containing inhibitors in vivo can be relatively low, necessitating further optimization in medicinal chemistry. Synthetic strategies, including direct *N*-substitution, acylation of appropriate *N*-O derivatives, and direct oxidation of the corresponding amide, have been employed to prepare a wide range of biologically active hydroxamic acids.

**Figure 15:**
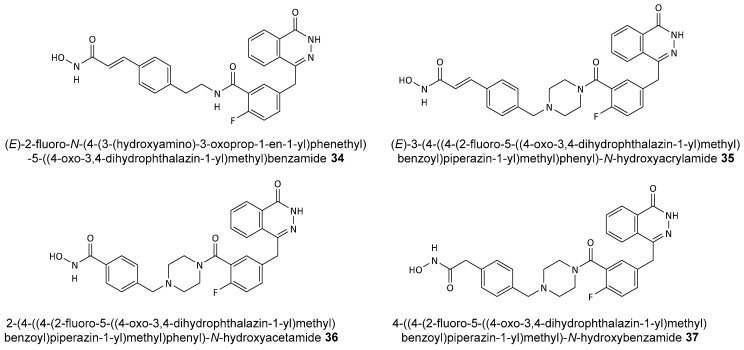
Structures of olaparib-derived dual PARP and HDAC inhibitors.

Hydroxamic acid derivatives have logically been explored for the design of inhibitors aimed at both enzymes. Yuan et al. reported the synthesis of hydroxamic acid derivatives of olaparib (**34–37**) (see Figure 15), demonstrating dual inhibition of PARP and HDAC with significant antitumor activity [93].

**Figure 16:**
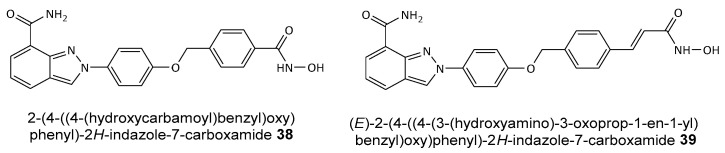
Structures of proposed hybrid PARP1/2 and HDAC6 inhibitors.

In recent years, there has been growing interest in developing multifunctional hybrid anticancer agents **38** and **39** (see Figure 16), including dual inhibitors that target both PARP1/2 and HDAC6 [42]. These dual inhibitors have demonstrated high cytotoxicity, though often non-selective, potentially due to their ability to chelate intracellular metal ions. Enhanced activity is typically observed in *N*-OH species when the nitrogen atom is not part of a cyclic structure, whereas non-selective cytotoxicity is less common in bioactive heterocycles containing endocyclic *N*-OH (*N*-OR) groups.

In a recent study by Bondar et al. [94], *N*-hydroxy derivatives of benzamide and other aryl hydroxamic acids, along with their *O*-substituted analogs, were explored as potential dual PARP1 and HDAC inhibitors. These compounds have not been systematically studied as PARP1 inhibitors to date. The aim was to evaluate their potency using both computational methods and experimental data, develop optimized synthetic pathways, and identify potential drug candidates.

The synthetic pathway involved transforming *N*-(benzyloxy)benzamides into phenanthridinones through reactions with unsubstituted iodobenzene, halophenols, or aryl iodides with strong electron-withdrawing groups. The resulting compounds included analogs of PJ34 **26**, a well-known PARP inhibitor, modified with *N*-OBn and *N*-OH moieties. These analogs were selected for molecular docking and dynamics simulations to predict their binding affinities and interactions with the catalytic domain of PARP1.

**Figure 17:**
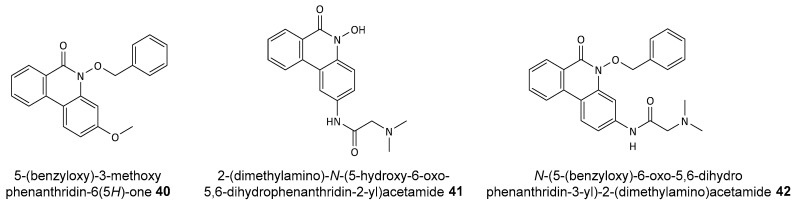
Structures of designed phenanthridinone-based PARP1 and HDAC1 inhibitors.

The selected compounds, labeled **40**, **41**, and **42** (see Figure 17), were tested for their ability to inhibit PARP1 activity using a PARP/Apoptosis Universal Colorimetric Assay Kit. The dose-dependent inhibition of PARP1 catalytic activity was observed, with IC_50_ values of 1.36 µM for **40**, 494.8 nM for **42**, and 48.9 nM for **41**, indicating a remarkable inhibitory effect, particularly for **41** and **42**.

Additionally, the hydroxamic acids, known HDAC inhibitors, were assessed for their HDAC1 inhibitory activity using a fluorogenic assay. Compound **41** showed an IC_50_ of 4.2 nM, confirming its potential as an HDAC inhibitor. This dual inhibition of PARP1 and HDAC1 by *N*-OH phenanthridinones contrasts with the selective PARP1 inhibition demonstrated by *N*-OBn derivatives, suggesting the need for further investigation.

The dual inhibition of PARP1 and HDAC1 by these novel hydroxamic acid derivatives underscores their potential as potent anticancer agents. The structural modifications, particularly the introduction of the dimethyl glycine moiety, appear to enhance anticancer activity. The balance of hydrophobic and hydrogen bond interactions within the catalytic pocket of PARP1 plays a critical role in determining binding affinity and specificity. This study presents one of the first experimental evidence of hydroxamic acids as dual inhibitors of PARP1 and HDAC, highlighting their potential for developing new anticancer therapies. Future work will focus on optimizing these compounds for enhanced selectivity and reduced toxicity, aiming to create more effective and safer therapeutic options.

## 10. Problems of Using Existing PARP1 Inhibitors

Despite their potential, existing PARP1 inhibitors face several significant challenges. Firstly, compounds that inhibit NAD+ binding often lack specificity and can block other enzymatic pathways involving NAD+, leading to high toxicity. This off-target effect stems from the competition with NAD+ for binding sites, which can result in unwanted inhibition of other NAD+-dependent enzymes and cellular processes, thereby increasing the risk of adverse effects [60]. Secondly, the use of enzymatic PARP1 inhibitors has been shown to activate virus replication. This is particularly problematic for patients infected with viruses such as the human T-cell leukemia virus (HTLV) or Kaposi’s sarcoma-associated virus (KSHV). In these cases, PARP1 inhibitors can exacerbate viral replication and disease progression, making them contraindicated for such patients [56]. Thirdly, the long-term safety of PARP1 inhibitors remains uncertain. Tumor cells can rapidly develop resistance to these drugs when used as long-term monotherapy. Resistance mechanisms include the restoration of homologous recombination repair through secondary mutations, upregulation of drug efflux pumps, and changes in drug target binding affinities. These adaptations by cancer cells reduce the effectiveness of PARP1 inhibitors over time, necessitating the development of combination therapies or novel inhibitors to overcome resistance [10].

These challenges highlight the need for ongoing research to improve the specificity, safety, and efficacy of PARP1 inhibitors, as well as to develop strategies to mitigate viral activation and resistance development in cancer therapy. The story of iniparib (BSI-201) **12** serves as a good example of the challenges faced by PARP inhibitors. Initially, iniparib **12** showed promising results in early trials, which led to significant excitement about its potential as a treatment for metastatic triple-negative breast cancer (TNBC). However, the Phase III clinical trial was ultimately discontinued due to a lack of improvement in patient outcomes [10].

Several factors contributed to the failure of iniparib. Firstly, there was insufficient preclinical testing. The initial studies did not adequately explore the drug’s pharmacodynamics and pharmacokinetics, leading to an incomplete understanding of its effectiveness and potential side effects in humans [56]. Secondly, there was a fundamental misunderstanding of the drug’s mechanism of action. Unlike other PARP inhibitors, iniparib was later found not to function as a true PARP inhibitor. Instead, it appeared to work through other, less well-understood mechanisms. This misunderstanding likely contributed to the observed lack of efficacy in the Phase III trial.

Other third-generation PARP inhibitors previously approved by the FDA encountered difficulties in their clinical use. Olaparib **1** is used for advanced ovarian cancer, early or metastatic breast cancer, metastatic pancreatic cancer, and metastatic prostate cancer [95]. Rucaparib **2** treats BRCA-positive metastatic castration-resistant prostate cancer and is used for the maintenance of ovarian, fallopian tube, or primary peritoneal cancer after a response to platinum-based chemotherapy [96].

Niraparib **3** is approved for maintenance in advanced ovarian, fallopian tube, or primary peritoneal cancer after a response to platinum-based chemotherapy [97]. Talazoparib **14** treats germline BRCA-mutated, HER2-negative breast cancer [98].

By October 2022, the manufacturers of rucaparib **2**, olaparib **1**, and niraparib **3** voluntarily withdrew their approvals for third- and fourth-line management of ovarian cancer due to safety concerns [99]. Recent studies showed negative effects on overall survival (OS) for these indications. In November 2022, the FDA limited rucaparib **2**’s use to second-line maintenance in BRCA-mutated patients and restricted niraparib to those with germline BRCA mutations [100].

Research suggests PARP inhibitors might enhance sensitivity to immune checkpoint inhibitors (ICIs) by affecting the tumor microenvironment and upregulating PD-L1 levels, which interact with PD-1 pathways [44].

The case of iniparib **12** and other third-generation PARP inhibitors underscores the importance of thorough preclinical testing and a deep understanding of a drug’s mechanism of action before proceeding to large-scale clinical trials. It also highlights the complexity of developing effective PARP inhibitors and the need for continuous research to address these challenges. To address these issues, future research aims to develop PARP inhibitors targeting other functional domains of PARP proteins, such as DNA-binding and transcriptional activities [10]. These new inhibitors could offer higher specificity and reduced side effects compared to current enzymatic inhibitors.

## 11. Perspectives in Greener Synthesis of PARP Inhibitors

Currently, the predominant methods for producing Active Pharmaceutical Ingredients (APIs) are through chemical syntheses [101,102,103], although some APIs can be derived from partially natural sources [104]. Unfortunately, these chemical processes are often not sustainable due to the extensive use of toxic and/or flammable organic solvents, which have caused considerable environmental harm over recent decades. It is estimated that around 85% of waste by mass in organic reactions is due to the use of these solvents [105]. Despite significant efforts to develop environmentally friendly alternative solvents [106], the processes still rely heavily on fossil-derived materials and require substantial energy for solvent production, purification, and recycling [107]. The Twelve Principles of Green Chemistry were established to address these issues [108,109] and have been widely developed over the last two decades [110].

In 2015, the United Nations adopted the Sustainable Development Goals (SDGs) to create a more sustainable and equitable future by 2030 [111]. These goals are particularly relevant to drug discovery: (1) SDG 3: Good Health and Well-being emphasizes the need for developing new medicines to improve global health, while (2) SDG 12: Responsible Consumption and Production encourages the adoption of eco-friendly practices in drug manufacturing to reduce waste and environmental impact; (3) SDG 6: Clean Water and Sanitation highlights the importance of managing water resources responsibly in drug production to prevent pollution. By aligning with these SDGs, the pharmaceutical industry is called to develop drugs that not only address health needs but also protect the environment and support global sustainability [112,113]. This approach ensures that drug development and production are both effective in improving health and responsible in their impact on the planet.

Today, the development of sustainable synthetic processes for new drug candidates is becoming a priority for pharmaceutical companies [114]. The process mass intensity (PMI) is an important metric of the impact of a synthetic route on chemical resources, cost, and sustainability [115,116,117]. It has been used for over 15 years in the pharmaceutical industry as the key metric for progress towards more sustainable manufacturing [118] and is currently the most comprehensive metric for measuring the resource usage impact of the synthetic chemistry processes used in small-molecule API manufacturing, quantifying the additional resource requirements to drive less resource-intensive and therefore more sustainable practices [119,120]. Recently, the International Union of Pure and Applied Chemistry (IUPAC) recognized mechanochemistry as one of the top 10 emerging technologies in chemistry, noting its potential for sustainable development and transformative impact on the field [121]. Mechanochemistry, which uses mechanical forces to drive chemical transformations, has a long history, initially benefiting fields such as inorganic chemistry and materials science [122]. In recent years, synthetic organic chemistry has increasingly adopted mechanochemistry to eliminate the need for solvents, thus aligning with sustainable practices and substantially lower PMI [123]. Mechanical forces are typically applied using equipment such as mortar and pestle, ball mills, or extruders. More recently, reactive extrusion has been introduced as a continuous flow technique for handling solids and concentrated mixtures. This advancement has expanded the range of organic molecules that can be synthesized mechanochemically, supporting the growth of the mechanosynthetic toolkit. This method aligns with the Green Chemistry principle of waste prevention [124], significantly reducing the amount of solvent required for reactions and purification. Mechanochemistry often enhances the overall efficiency of reactions, simplifying purification processes and reducing the need for solvent-intensive column chromatography [125].

**Figure 18:**
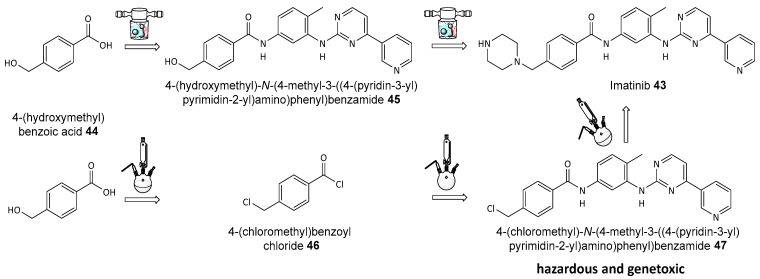
The traditional (**bottom**) and mechanochemical (**top**) synthesis of imatinib.

Imatinib **43**, an essential anticancer drug listed by the World Health Organization and commercially known as Gleevec (or Glivec), plays a critical role in treating chronic leukemia and gastrointestinal tumors, making these advancements particularly impactful for both manufacturing efficiency and patient safety. The mechanochemical synthesis of imatinib using ball-milling (see Figure 18) offers significant advances by reducing the number of steps from three to two, avoiding genotoxic intermediates, and improving safety and sustainability compared to traditional methods, which require high temperatures and toxic solvents [126].

One particularly promising application of mechanochemistry is in the synthesis of PARP inhibitors. Traditional methods for synthesizing PARP inhibitors involve multiple steps and the use of large volumes of solvents, which pose significant environmental and safety challenges [127,128]. Mechanochemical approaches offer a more sustainable and efficient pathway, minimizing the need for solvents and potentially reducing the number of synthetic steps required. This not only aligns with the principles of Green Chemistry but also enhances the scalability and accessibility of these important therapeutic agents.

Another approach to optimize drug development is flow chemistry, which offers numerous advantages, significantly enhancing the efficiency, safety, and sustainability of chemical synthesis [129]. One of its primary benefits is the precise control over reaction conditions, such as temperature, pressure, and mixing, leading to improved reaction yields and reproducibility. Flow chemistry enables continuous processing, reducing the need for large-scale batch reactors and minimizing downtime, thereby increasing overall productivity. This mode of operation also allows for safer handling of hazardous chemicals and exothermic reactions by maintaining small reactor volumes and enhancing heat dissipation [130]. Furthermore, flow chemistry supports process intensification, leading to shorter reaction times and lower energy consumption.

**Figure 19:**
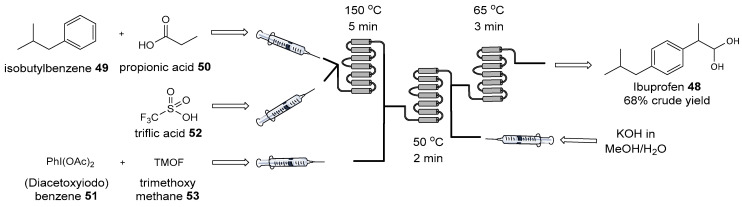
Continuous-flow synthesis of ibuprofen.

The versatility of this approach was clearly proven by the continuous-flow synthesis of ibuprofen **42** (see Figure 19), the most widely used painkiller in Europe, by enhancing efficiency and reducing waste through key synthetic reactions such as Friedel–Crafts acylation, 1,2-aryl migration, and saponification, all performed without intermediate purification steps [131]. The streamlined nature of flow setups often eliminates the need for extensive purification steps, such as chromatography, reducing waste generation and environmental impact. The scalability of flow processes, from laboratory to industrial scales, makes them highly attractive for pharmaceutical manufacturing, fine chemicals production, and other sectors requiring high-throughput and sustainable chemical synthesis [132].

**Figure 20:**
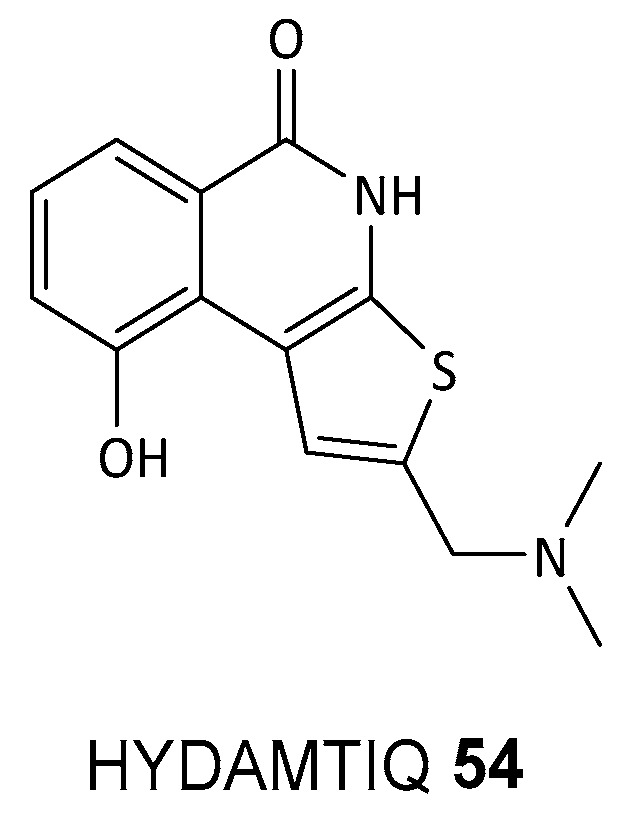
Structure of flow chemistry prepared PARP1/2 inhibitor HYDAMTIQ **54**.

The continuous flow synthesis of the PARP1/2 inhibitor HYDAMTIQ **54** (see Figure 20) illustrates these advantages [133]. It has shown potential as a neuroprotective agent by reducing brain infarct volumes and leukocyte infiltration in brain ischemia models. Additionally, it decreases lung PARP activity, alleviates allergen-induced cough and dyspnea, and reduces bronchial hyperreactivity, indicating its use in chronic lung inflammation, airway damage, and asthma. HYDAMTIQ **54** also shows promise in oncology for treating ovarian, breast, prostate, and pancreatic cancers, as well as glioblastoma multiforme [134].

The continuous flow synthesis process significantly improves efficiency, safety, and sustainability. The precise control over reaction conditions results in a higher yield of 55% and ≥97% purity compared to 23% in batch processing. Safety is enhanced by managing hazardous reactions in small volumes, while productivity is increased by reducing downtime and eliminating extensive purification steps. Waste generation was significantly reduced, with the E-factor dropping from 4172 to 488. These improvements make flow chemistry highly attractive for pharmaceutical manufacturing and other high-throughput industries.

Mechanochemistry and flow chemistry offer promising advantages for sustainable drug development of PARP inhibitors. These approaches align with green chemistry principles, enhancing efficiency, safety, and eco-sustainability. Mechanochemistry eliminates the need for solvents and improves reaction efficiency, making it suitable for mass-scale implementation. Flow chemistry, with precise control and continuous processing, reduces waste and boosts productivity.

## 12. Conclusions

To conclude, the PARP inhibitors represent a significant advancement in oncology, particularly for tumors with defective DNA repair pathways. As research progresses, new strategies and inhibitors will likely emerge, addressing current challenges and expanding the therapeutic potential of PARP inhibition. The development of inhibitors targeting additional PARP activities beyond the catalytic domain holds promise for more effective and less toxic cancer therapies. In conclusion, while existing PARP inhibitors have demonstrated potential as anticancer agents, challenges such as specificity, toxicity, and resistance remain. Future research should focus on developing inhibitors that target other functional domains of PARP proteins and exploring combination therapies to enhance their efficacy. The continued advancement of PARP inhibitors offers hope for more effective cancer treatments, particularly for patients with tumors characterized by defective DNA repair mechanisms.

Poly(ADP-ribose) polymerase (PARP) inhibitors have become an important tool in cancer treatment, especially for patients with certain types of tumors that have problems repairing their DNA, such as those with BRCA mutations. Over the years, the development of these drugs has progressed significantly. Early versions were less targeted and effective, but the latest generations are much more precise and powerful, making them key components in the treatment of several cancer types.

The path from the first versions of PARP inhibitors to the most recent ones has led to drugs that not only work better but also offer more personalized treatment options. These advances have improved the survival rates and quality of life for many cancer patients by allowing treatments to be tailored to the specific deficiencies of their tumors.

However, there are still challenges that need to be addressed. One major problem is that some tumors can develop resistance to PARP inhibitors, finding ways to bypass the effects of the drugs. Understanding how this resistance happens is crucial for developing new versions of PARP inhibitors or combining them with other treatments to make them more effective.

Another concern is that while modern PARP inhibitors are more focused on their targets, they can still affect other functions in the body, leading to side effects. This highlights the need for ongoing research to create even more precise drugs that minimize these unwanted effects.

Combining PARP inhibitors with other treatments, such as chemotherapy, radiation, or immune therapies, shows great promise. These combinations could make the drugs even more effective, especially for tumors that are difficult to treat.

Looking ahead, the development of PARP inhibitors will likely focus on a few key areas. One is creating drugs that can target multiple pathways in cancer cells at once, which could enhance their effectiveness and reduce the chance of resistance. Another is making the production of these drugs eco-friendly, which aligns with the broader push for sustainability and green chemistry principles in drug development.

Additionally, researchers are exploring the use of PARP inhibitors beyond cancer, in conditions such as neurodegenerative diseases, heart problems, and inflammatory conditions. Expanding their use in these areas could provide new treatment options for a variety of diseases.

In summary, while PARP inhibitors have made significant strides in cancer treatment, there is still more to be done. Continued innovation and research are essential to fully unlocking their potential, not just in cancer therapy but possibly in treating other diseases as well. The future of PARP inhibitors looks promising, with the potential to greatly improve patient care across a wide range of health conditions.

## Data Availability

All the data gathered for this study is available in the article.

## Supplementary Materials

The following supporting information can be downloaded at: https://www.mdpi.com/article/10.3390/biom14101269/s1: Figure S1. Structures of the compounds and their corresponding codes as referenced in this work.

## Abbreviations

BER—base excision repair; CRISPR—clustered regularly interspaced short palindromic repeats; CT—clinical trials/studies; DHB—L-threitol-1,4-bismethanesulfonate; DNA—DSB—double-strand breaks; FDA—U.S. Food and Drug Administration; HR—homologous recombination repair; MMR—mismatch repair; NAD+/NADH—nicotinamide adenine dinucleotide; NHEJ—non-homologous end joining; NER—nucleotide excision repair; PARP—poly(ADP-ribose) polymerase; PHD—6(5*H*)-phenanthridinone; PLD—potentially lethal damage; PMI—process mass intensity; ROS—reactive organic species; SSB—single-strand breaks; TMZ—temozolomide; TOP1—topoisomerase I.
