# Supplementary material for: Poly(ADP-Ribose) Polymerase (PARP) Inhibitors for Cancer Therapy: Advances, Challenges, and Future Directions
Source: Biomolecules. 2024 Oct 9;14(10):1269. doi: 10.3390/biom14101269 (PMC11506039; doi:10.3390/biom14101269)
Supplement: Supplementary file 1 [file biomolecules-14-01269-s001.zip › biomolecules-3212547-supplementary.pdf]

Electronic Supporting Information

Poly(ADP-Ribose) Polymerase (PARP) Inhibitors for Cancer Therapy: Advances, Challenges, and Future Directions

Denys Bondar[1] and Yevgen Karpichev[1]

[1] Department of Chemistry and Biotechnology, Tallinn University of Technology (TalTech), Tallinn, Estonia

Table of Content

Figure S1. Structure of the compounds and their corresponding codes as referenced in this work………S2

Figure S1. Structure of the compounds and their corresponding codes as referenced in this work.

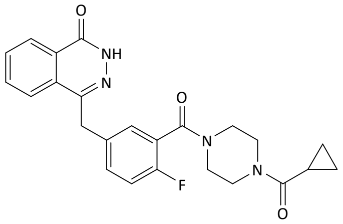

Olaparib **1**

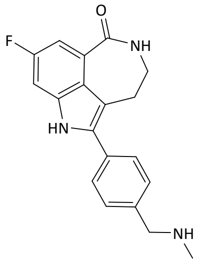

Rucaparib **2**

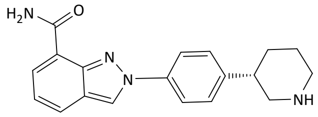

Niraparib **3**

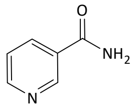

Nicotinamide **4**

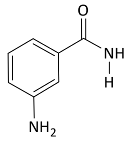

3-aminobenzamide **5**

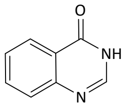

Quinazoli*n*one 6

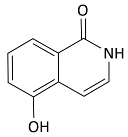

5-hydroxy isoquinolin-1(2*H*)-one **7**

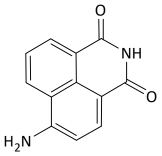

6-amino-1*H*-benzo[*de*] isoquinoline-1,3(2*H*)-dione **8**

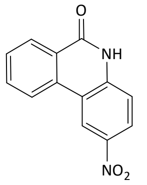

2-nitrophenanthridin -6(5*H*)-one 9

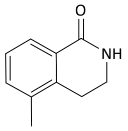

5-methyl-3,4-dihydro isoquinolin-1(2*H*)-one **9**

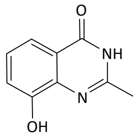

8-hydroxy-2-methyl quinazolin-4(3*H*)-one **11**

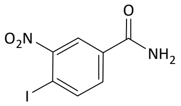

Iniparib **12**

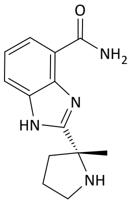

Veliparib **13**

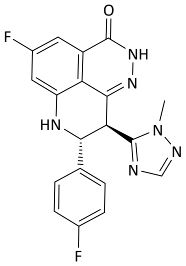

Talazoparib **14**

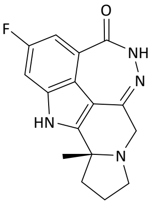

Pamiparib **15**

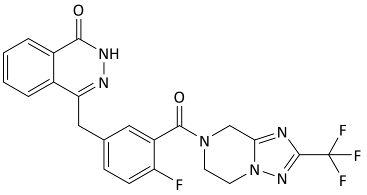

Fuzuloparib **16**

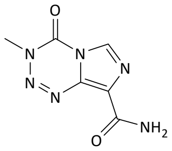

Temozolomide **17**

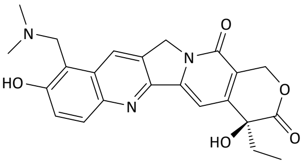

Topotecan **18**

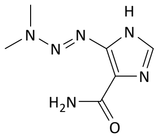

Dacarbazine **19**

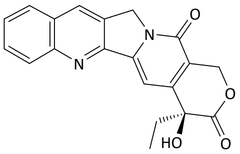

Camptothecin **20**

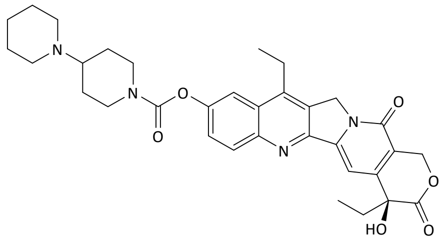

Irinotecan **21**

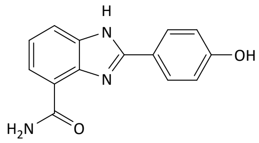

2-(4-hydroxyphenyl)-1*H*-benzo[*d*] imidazole-4-carboxamide **22**

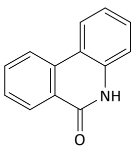

6(5*H*)-phenanthridinone **23**

Figure S1. (*continued*)

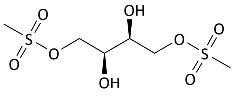

L-threitol-1,4-bis methanesulfonate **24**

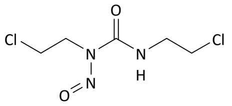

carmustine **25**

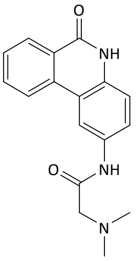

PJ34 **26**

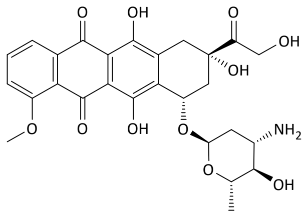

Doxorubicin **27**

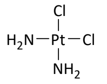

Cisplatin **28**

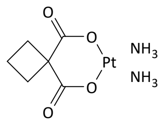

Carboplatin **29**

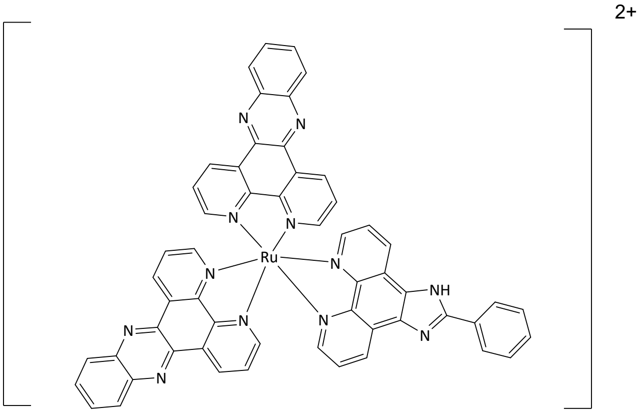

[Ru(dppz)2(PIP)]$^{2+}$ **30**

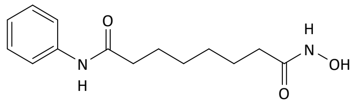

Vorinostat **31**

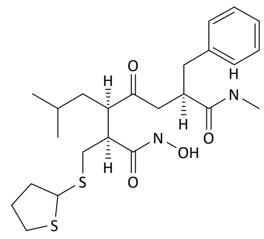

Batimastat **32**

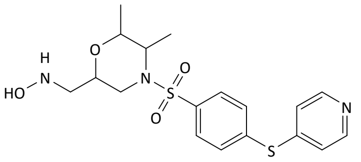

Prinomastat **33**

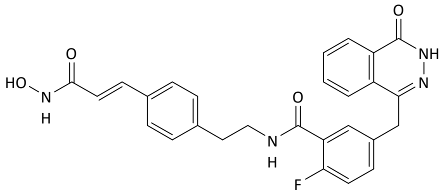

(*E*)-2-fluoro-*N*-(4-(3-(hydroxyamino)-3-oxoprop-1-en-1-yl)phenethyl) -5-((4-oxo-3,4-dihydrophthalazin-1-yl)methyl)benzamide **34**

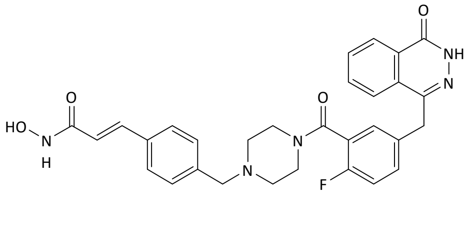

(*E*)-3-(4-((4-(2-fluoro-5-((4-oxo-3,4-dihydrophthalazin-1-yl)methyl) benzoyl)piperazin-1-yl)methyl)phenyl)-*N*-hydroxyacrylamide **35**

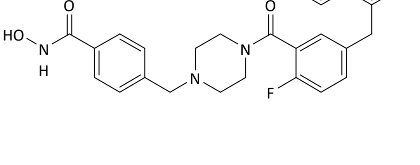

2-(4-((4-(2-fluoro-5-((4-oxo-3,4-dihydrophthalazin-1-yl)methyl) benzoyl)piperazin-1-yl)methyl)phenyl)-*N*-hydroxyacetamide **36**

Figure S1. (*continued*)

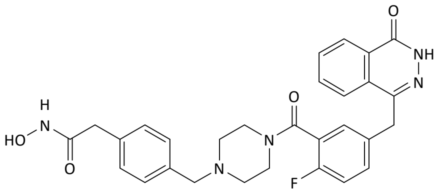

4-((4-(2-fluoro-5-((4-oxo-3,4-dihydrophthalazin-1-yl)methyl) benzoyl)piperazin-1-yl)methyl)- *N*-hydroxybenzamide **37**

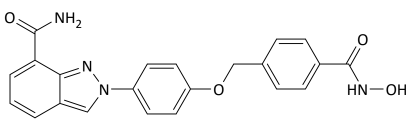

2-(4-((4-(hydroxycarbamoyl)benzyl)oxy) phenyl)-2*H*-indazole-7-carboxamide **38**

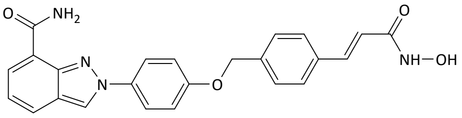

(*E*)-2-(4-((4-(3-(hydroxyamino)-3-oxoprop-1-en-1-yl) benzyl)oxy)phenyl)-2*H*-indazole-7-carboxamide **39**

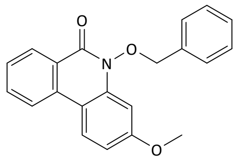

5-(benzyloxy)-3-methoxy phenanthridin-6(5*H*)-one **40**

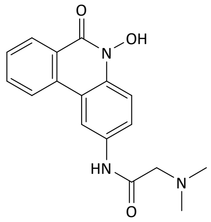

2-(dimethylamino)- *N*-(5-hydroxy-6-oxo-5,6-dihydrophenanthridin-2-yl)acetamide **41**

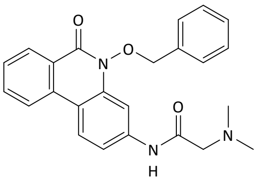

N-(5-(benzyloxy)-6-oxo-5,6-dihydro phenanthridin-3-yl)-2-(dimethylamino)acetamide **42**

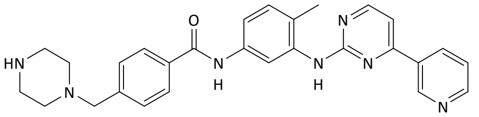

Imatinib **43**

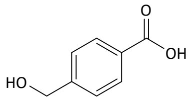

4-(hydroxymethyl)benzoic acid **44**

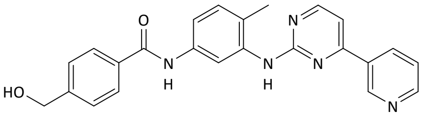

4-(hydroxymethyl)- *N*-(4-methyl-3-((4-(pyridin-3-yl) pyrimidin-2-yl)amino)phenyl)benzamide **45**

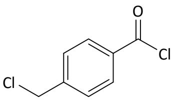

4-(chloromethyl)benzoyl chloride **46**

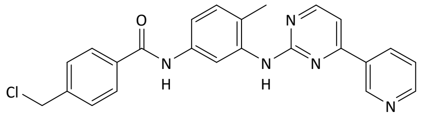

4-(chloromethyl)- *N*-(4-methyl-3-((4-(pyridin-3-yl) pyrimidin-2-yl)amino)phenyl)benzamide **47**

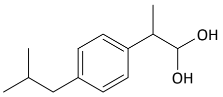

Ibuprofen **48**

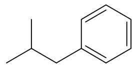

isobutylbenzene **49**

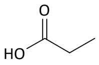

propionic acid **50**

PhI(OAc)$_2$

(Diacetoxyiodo) benzene **51**

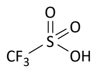

triflic acid **52**

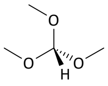

trimethoxy methane **53**

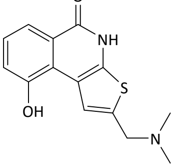

HYDAMTIQ **54**
